# Etiology, Modalities of Zygomaticomaxillary Complex Fracture, open reduction and fixation.

**DOI:** 10.4317/jced.57445

**Published:** 2021-03-01

**Authors:** Rohit, Vishal, Virendra-Kumar Prajapati, Ajoy-Kumar Shahi, Om Prakash, Subia Ekram

**Affiliations:** 1 Tutor. Department of Oral and Maxillofacial Surgery. Dental Institute. Rajendra Institute of Medical Sciences; 2 Professor. Head of Department. Department of Oral and Maxillofacial Surgery. Dental Institute. Rajendra Institute of Medical Sciences; 3 Associate Professor. Department of Oral and Maxillofacial Surgery. Dental Institute. Rajendra Institute of Medical Sciences; 4 Assistant Professor. Department of Oral and Maxillofacial Surgery. Dental Institute. Rajendra Institute of Medical Sciences

## Abstract

**Background:**

Zygomatic complex fracture is second most common mid face fracture and frequent amongst the maxillofacial trauma. Fracture pattern ranges from simple to comminuted and from minimally displaced to severely displaced depending on various factors.

**Material and Methods:**

98 patients with zygomaticomaxillary complex fracture reporting during December 2017 to January 2020 were included in the study. On the basis of radiographic evaluation and computerized tomography scan (CT scan) with 3D reconstruction severity of fracture was assessed and different treatment options were selected.

**Results:**

Road traffic accident accounted as the leading cause of fracture (57.1%) followed by self-fall (16.3%), interpersonal violence (12.3%). Reduction and semi rigid fixation was done in (83.7%), in which 1-point fixation in (22.9%), 2-point fixation in (42.4%) and 3-point fixation in (18.4%). Rest 16.3 % of the cases were managed conservatively since they had minimal displacement.

**Conclusions:**

Road traffic incident was the main etiology in our study and younger age group patients were more involved. Occipitomental radiograph and computerized tomography scan (CT scan) were used to confirm the diagnosis and to determine the severity of displacement of zygomatic fracture on the basis of which treatment options were decided.

** Key words:**Incidence, etiology and management zygomaticomaxillary complex fracture

## Introduction

Zygoma is a strong buttress of the lateral portion of middle third of facial skeleton and it significantly contribute to the strength and stability of mid face. Zygomatic fracture is one of the most common and frequent among the maxillofacial trauma, due to its prominence which predisposes it to bear the brunt of facial injuries. Moderate to severly displaced zygomatic fracture can significantly alter the structure, function, and appearance of midface. Fracture pattern ranges from simple to comminuted and from minimally to severely displaced depending on the nature and impact of the injury. Zygomatic fracture is traditionally referred as “tripod” but actually it involves disruption at four sites depending on the impact and severity: lateral orbital rim, inferior orbital rim, zygomaticomaxillary buttress and the zygomatic arch. These fracture are the second most fracture of the face after nasal injuries (1). Epidemiology of maxillofacial fracture varies between populations, particularly with regard to the incidence, demographic distribution of the fractures, aetiology and types vary due to environmental, socioeconomic, cultural and lifestyle differences (2). Radiological evaluation such as computerized tomography scan with reconstruction, occipitomental view, submentovertex view clearly detects the fracture and its displacement. Various treatment plan has been modulated for zygomaticomaxillary complex fracture depending on the degree of displacement of the zygomatic bone which ranges from simple conservative management to open reduction and multiple point of exposure and fixation (3). Isolated zygomatic bone fracture with minimal displacement can be managed with closed reduction without fixation (3).

## Material and Methods

This study was carried out at Dental institute, Rajendra Institute of Medical Sciences (RIMS), Ranchi. All patients who had sustained zygomaticomaxillary complex fracture reporting to the Department of Oral and Maxillofacial surgery at Dental institute and emergency OPD at RIMS Hospital Ranchi were included in the study. Study duration was from December 2017 to January 2020. Patients included in the study were 98. Exclusion criteria was decided which are as follow.

Exclusion criteria:

• Head injury patients with severe brain parenchyma lesion.

• Orbital fractures, where additional procedure is required for reconstruction of orbital floor 

• Fractures more than 6 weeks old 

• Patients with systemic disorder where surgery was contraindicated.

The criteria used to determine the need of surgical correction consisted of both clinical and radiological assessment. CT scan with 3-D reconstruction was used for radiological evaluation Depending on the different patterns of zygomatic bone fracture which ranges from simple fracture to comminuted and from minimally displaced to severely displaced, treatment options were decided. Undisplaced zygomatic bone fracture were treated conservatively and were recalled for regular follow up while displaced and comminuted fractures of zygoma were surgically corrected. Different surgical approaches were used depending on the degree of displacement of zygoma. Operative procedure involved open reduction and internal fixation using 1.5 mm / 2mm titanium mini plates and mesh with multiple points of exposure and fixation at 1-point, 2-point and 3-point fixation was done under Local and General Anesthesia.

## Results

During two years of study from December 2017 to January 2020 which included follow up period also, 98 patients with ZMC fracture were treated, of which 72.4% (n=71) male and 27.6% (n=27) females with Male: Female ratio of 2.6:1 

In the population studied, road traffic accident was found to be the most common etiology of the zygomatic bone fracture accounting for 57.1% (n=56) of the cases, followed by accidental self-fall representing 16.3% (n=16) of the cases, assault (inter personal violence) 12.3% (n=12) and work related injuries accounting for 7.1 % (n=7), sports injury (n=3) 3.1% and animal bite (bear bite injury) (n=4) 4.1% (Table 1).

**Table 1:**
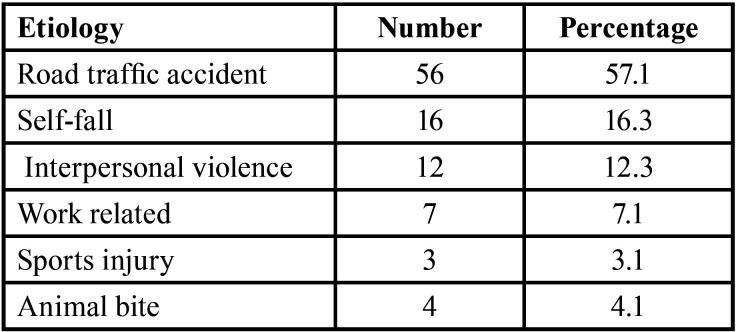
Different etiology Zygomatic bone fracture.

Data regarding clinical presentation during the initial examination of the patients were recorded. Patients presented with circumorbital ecchymosis and perioorbital edema and subconjunctival ecchymosis were the most common sign followed by chemosis, flattening of the cheek, step deformity, infraorbital nerve paresthesia,visual acuity was though limited to few cases while evisceration was seen in (n=2) patient.

Study showed that zygomatic bone was fractured at single process in 36.8% (n=36) and more than one process was involved in 63.2% (n=62). In patients with single process fracture zygomaticomaxillary buttress (ZMB) was most commonly involved accounting for 14.3% (n=14), followed by infraorbital rim (IOR) 10.7% (n=10), isolated zygomatic arch 8.2% and Frontozygomatic (FZ ) 4.1% (Table 2).

**Table 2:**
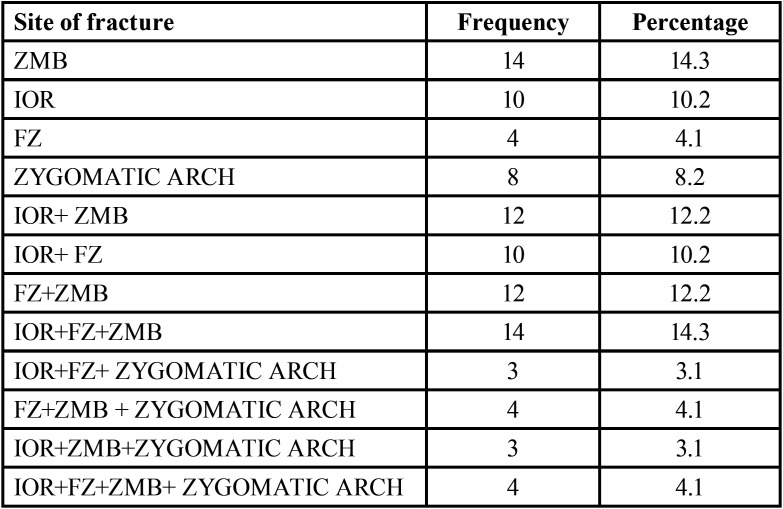
Different Sites of zygomatic bone fracture.

When zygomatic bone was fractured at more than one process, fracture at two processes was found in 34.60% patients, Three process fractures (tripod) were reported 24.5% of cases and in 4.1% cases involved zygomatic buttress, orbital rim, frontozygomatic suture and zygomatic arch.

Out of 98 patients, 16.3% (n=16) were diagnosed with undisplaced zygomatic fracture who did not require any surgical intervention and were managed conservatively with periodic follow ups. In 83.7% (n=82), open reduction and internal fixations were carried out under local and general anesthesia .Various surgical approaches were made to access the fractured ends such as subcilliary, lateral brow, intraoral vestibular incision or through existing laceration, and Hemicoronal approach. Depending on the severity of the injury, degree of displacement and stabilization required after reduction, fixation was done at either1-point, 2-point or 3-point. 1-point fixation was done 22.9% (n=22) of the cases; 2-point fixation was done 42.4% (n=42) of the cases and 3-point fixation 18.4% (n=18) of the cases (Table 3).

**Table 3:**
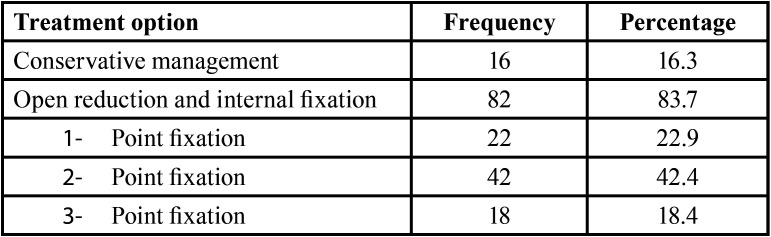
Different treatment options for Zygomatic bone fracture.

In 1-point fixation cases ZMB was fixed in 63.7% (n=14) while in 2-point fixation FZ and ZMB was fixed in 57.1% (n=24) cases and three point fixation was done in 83.3% (n=15) (Table 4).

**Table 4:**
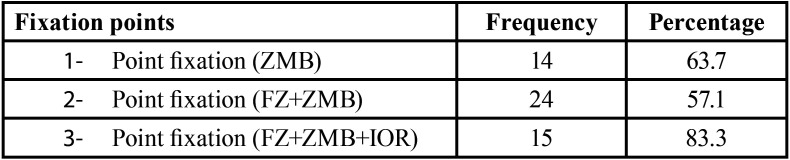
Different fixation point for 1-point, 2-point & 3-point for Zygomatic bone fracture.

During the period of postoperative follow up, no cases were encountered with incidence of mobility of fractured segments. Complications such as facial asymmetry, occlusal discrepancies, persistent infra orbital sensory nerve disturbance was seen in few cases.

## Discussion

Zygomatic bone contribute significantly to the strength and stability of mid face and acts as a buttress. It is considered to be the foundation for person aesthetic appearance by setting midfacial width and providing prominence to cheek. It can best be describes as “Terapod” as it maintain four points of articulation with frontal, temporal, maxillary bone and greater wing of sphenoid. ZMC fracture represent the second most common type of facial bone fracture after nasal bone fracture due to its prominence (4). Our study recorded that more males than females sustained zygomatic bone fracture (2.6:1) and similar finding were found in other studies like Ozemen *et al*. (5,6) (3.2:1), Chowdhury *et al*. (1) (5.2:1).

Age group most commonly involved in our study was 3rd decade, reason could be greater social involvement of young adult male. Road traffic accident was the most common cause of the zygomatic bone fracture in present study, especially two wheeler motor bike accident had a high frequency, Similar high percentage of road traffic accidents were reported by Chowdhury and Menon (1) 86.20%, Fasola *et al.* (2) 81.6% however, Kovacs *et al.* (7) 46.2%, reported assault as the leading cause of zygomatic fracture. Interestingly Sulliven STO *et al.* (8) reported Sports injury as 27.5%. Our study also reported four case of open and comminuted ZMC fracture, etiology being animal (Bear) bite injury. The etiology of facial fractures has changed over decades and they continue to do so (9).

The zygomatic bone provides height, width and projection to the face and forms a part of the bony orbit. ZMC fracture which is displaced inferiorly results an anti-mongoloid slant and accentuation of the supratarsal fold of the upper eyelid, may result in disturbed ocular functions, orbital shape and facial esthetics (10,11). Evaluation of a patient with a ZMC fracture included evaluation of bony injuries and status of surrounding soft. Visual acuity was ascertained and ophthalmological consultation was obtained in doubtful case. However in our study three patient presented with altered visual acuity both preoperative and two patient had evisceration.

Detailed history, clinical examination and palpation of was done in orderly fashion. Tenderness, a step off or discontinuity of the bony frame indicated possibility of fracture. The infraorbital nerve involvement was seen in majority of cases. Buccal vestibule ecchymosis and tenderness or disruption in the zygomatic buttress was elicited. Mandibular movement such as protrusion maximum mouth opening was evaluated to Figure out any impingement of the zygomatic process or the arch on the coronoid process of mandible.

Radiographic diagnosis of ZMC fracture was done using. CT scans (Axial and coronal view) with 3D applications of the mid face helped us to visualize and quantify malar eminence displacement in the anterior-posterior, medial-lateral, and superior- inferior dimensions (12).

Our study showed occurrence of single processes fracture in 28.6% and isolated zygomatic arch fracture in 8.2% of cases, 5.1% of the Patients who sustained isolated and inward displaced zygomatic arch fracture had restricted mouth opening, which was corrected by elevating the arch intraorally. Isolated fracture at zygomaticomaxillary buttress was seen in 14.3% followed by isolated fracture at Infra orbital rim which accounted for 10.2%More than one process fracture was seen in 63.2%, out of which 34.6% had two process fracture and tripod, three process fracture was seen in 24.5%. In 4.1% of cases zygomatic bone was separated from all the articulation. Zing *et al.* (13) reported tripod fracture in 51% of cases which is high as compared to this study. The majority of ZMC fractures are closed, displaced and non-comminuted, open and comminuted have lesser frequency and are seen mostly in severe traffic accident depending on magnitude of impact and vector. Zachariadest *et al.*. (14) defined in his literature that management of ZMC fracture depends on degree of displacement and the resultant esthetic and functional deficit. Depending on the intensity of impact, the fractures of the zygomatic complex could be isolated, single and undisplaced as seen in low energy impact cases or they could be displaced and rotated at one or more points around vertical and horizontal axis as seen in medium and high velocity injuries. Pull of the attached muscles, related to displacement of zygomatic process either enbloc or comminuted, makes the closed reduction of these fracture ineffective (15,16). Although it has been suggested that all displaced ZMC fracture require surgical intervention, conservative management is frequently employed in cases of minimal displacement, rotation and non-compliance of patient towards surgery. In this study 16.3% of the cases had undisplaced fracture with almost no displacement and they did not require any surgical treatment and were followed up over a variable period of time. We did not encountered any case with late displacement or rotation during follow up of these patients.

Proper reduction and fixation of displaced zygomatic fractures are essential to ensure proper healing and prevent post-operative complications. There is no need to address all the four articulation to achieve an acceptable reduction however, at least one, two or three articulations out of four must be addressed intra operatively to reduce these fractured segments accurately. Different treatment strategies for the treatment of zygomatic bone fracture were described in literatures, such as Elevation with hook, External pin fixation, Antral packing with gauge, Intraosseous wiring (17,14). All these procedures had their own advantage and disadvantages. The lack of directional control and factors like insufficient contact area, fracturing of bone in excessive tightening and healing by secondary intention were the problem areas in the management of ZMC fracture initially with wire osteosynthesis (18,10). Emergence of miniplates and screws which were malleable and miniaturized for maxillofacial fracture fixation resolved the problem associated with wire osteosynthesis.

In our study for exposure of the fractured site lateral brow incision was given for the reduction and fixation of fractured ends at FZ area, subcilliary approaches were used for exposure at infraorbital rim, and maxillary vestibular incision was used for reduction and fixation of ZMB region. In few cases fractured site was reached through existing laceration. We used hemicoronal approach for accurate reduction and fixation of comminuted and severely displaced zygomatic bone. One of the most controversial topics in maxillofacial trauma is how much fixation is enough to prevent post reduction displacement of the fractured ZMC (19,3).

In our study 83.7% cases were treated by open reduction and internal fixation using miniplates. One-point fixation was done in 22.9% of cases in which fixation at ZMB was done in 14cases followed by 3 cases at IOR margin and in 5 cases FZ was stabilized and fixed. Two-point fixation was done in 42.4% cases in which 24 cases were fixed at FZ and ZMB region, 8 cases at IOR and FZ and 10 case at IOR and ZMB region. Three -point fixation was carried out in 15 patients accounting for 18.4% Different point of fixation in our study was based on severity of displacement. Ji Heui kim *et al.* (20) concluded that one-point fixation at the ZMB through a gingivobuccal sulcus incision was effective for isolated fracture of zygoma without comminution of lateral orbital rim, because the ZMB plays a key role in withstanding contraction of the masseter muscle and supporting zygoma, rigid fixation at the ZMB is important in treatment of isolated zygomatic fracture. However, Champy *et al.* (21) and Mitchell *et al.* (22) in his study reported satisfactory results with a single point fixation of the zygomatic complex fracture at the FZ region.

In accordance with the Biomechanics of the facial skeleton as discussed by Rudderman and Mullen., fractured zygomatic segments has six possible direction of motion: translation across x, y, z axis and rotation about x,y,z axis (23). A miniplate fixed across the FZ suture will resist translatory movement and also rotation along an axis perpendicular to the plane of miniplate because of the width of the plate. At the same time, it will offer little resistance to rotation along the linear axis of plate. To improve the stabilization, an additional plate is to be applied in a manner where the weak axis of both the plane doesn’t coincide with a line connecting them (24,25,14). Davidson *et al.* stated that the two-point fixation using miniplate alone conferred a degree of stability comparable to most methods of three-point fixation regardless of the site in which the miniplates were applied (26). Two point fixation limits the translatory and rotational movement of stabilized moderately displaced ZMC fracture (26,11). The masseter muscle has often been implicated as a main cause of post reduction displacement of the fractured ZMC, because of inferior directional pull.

We did not encountered any evidence of displacement of fractured segments post stabilization and fixation regardless of the number of fixation device applied. There was neither any evidence of loosening of plates nor infection in the operated site. Apart from this on clinical evaluation postoperatively there was no evidence of movement of fixed fractured segments. Postoperative complication such as oedema, trismus, ocular complication gradually resolved over a period of time (27-30). Paresthesia at infraorbital region was seen in 16 patients even after a long follow up period, occlusal discrepancy was seen in patients who had severely displaced fracture.

## Conclusions

Different treatment protocol for the management of ZMC fractures begin with precise and expedient diagnosis that account for proper reduction of fractured segments to restore facial balance. The conflict still persist regarding accurate reduction, stabilization and fixation of zygomatic bone fracture. In our study fixation points were either 1-point, 2-point & 3-point depending on the displacement at the fractured sites. Two-point fixation was the most common, which was based on the severity and displacement of fractured end. Based on our experience and the facts generated from our study, a variety of methods can be used successfully to stabilize ZMC fracture which depends on the characteristics of the fracture and open reduction and internal fixation with miniplates is the most reliable modality providing three dimensional stability.
